# Infection, inflammation and hepatic encephalopathy from a clinical perspective

**DOI:** 10.1007/s11011-024-01402-y

**Published:** 2024-08-30

**Authors:** Yevedzo Ntuli, Debbie L. Shawcross

**Affiliations:** 1 School of Immunology and Microbial Sciences, Faculty of Life Sciences and Medicine, King’s College London, King’s College Hospital, 125 Coldharbour Lane, London, SE5 9NU UK; 2 Institute of Liver Studies, King’s College Hospital, Denmark Hill, London, SE5 9RS UK

**Keywords:** Ammonia, Cirrhosis, Covert hepatic encephalopathy, Overt hepatic encephalopathy

## Abstract

Hepatic encephalopathy (HE) is a syndrome that is associated with both acute and chronic liver injury. It manifests as a wide spectrum of neuropsychological abnormalities, ranging from subtle impairments in executive higher functions observed in cirrhosis, through to coma in acute liver failure. In acute liver failure, the central role of ammonia in the development of brain oedema has remained undisputed for 130 years. It latterly became apparent that infection and inflammation were profound determinants for the development of severe hepatic encephalopathy, associated with the development of cerebral oedema and intracranial hypertension. The relationship of the development of hepatic encephalopathy with blood ammonia levels in cirrhosis is less clear cut and the synergistic interplay of inflammation and infection with ammonia has been identified as being fundamental in the development and progression of hepatic encephalopathy. A perturbed gut microbiome and the presence of an impaired gut epithelial barrier that facilitates translocation of bacteria and bacterial degradation products into the systemic circulation, inducing systemic inflammation and innate and adaptive immune dysfunction, has now become the focus of therapies that treat hepatic encephalopathy in cirrhosis, and may explain why the prebiotic lactulose and rifaximin are efficacious. This review summarises the current clinical perspective on the roles of inflammation and infection in hepatic encephalopathy and presents the evidence base for existing therapies and those in development in the setting of acute and chronic liver failure.

## Introduction

Hepatic encephalopathy (HE) encompasses a spectrum of neurocognitive abnormalities caused by liver disease and/or portosystemic shunting. It manifests clinically with characteristic aberrations in cognitive, behavioural and motor functioning, as well as changes in consciousness, culminating in stupor and coma in its most severe form. HE is classified by aetiology into type A, B or C. Type A is caused by acute liver failure, most commonly caused by drug-induced injury (mainly paracetamol overdose), acute viral hepatitis or vascular pathology (Bernal et al. 2013). Its development in acute hepatic injury is a diagnostic feature of acute liver failure along with the presence of coagulopathy. Complications include cerebral oedema, raised intracranial pressure (ICP) and cerebral herniation, associated with high mortality rates (Clemmesen et al. 1999; Bernal et al. 2007; Karvellas et al. 2009; Shawcross and Wendon 2012). Type B is caused by portosystemic shunting in the absence of liver disease. Type C is associated with chronic liver disease and occurs across a spectrum of severity and is most simply classified into ‘covert’ and ‘overt’ stages, and historically by the Westhaven criteria from grades 1 to 4 (Galambos 1979; Ferenci et al. 2002; Vilstrup et al. 2014) (See Table 1). The development of HE in cirrhosis represents an acute decompensation event and is associated with significant morbidity and mortality (Bajaj et al. 2017b; Bohra et al. 2020). Theories about the pathogenesis of HE have historically placed ammonia metabolism at the centre. However, it is now widely recognised that infection and inflammation have a synergistic role in the development of HE. In liver disease, both are influenced by the interaction of gut microbiota with the intestinal epithelial barrier, dysfunction of which, causes a constant source of pathogenic material from the intestinal lumen to stimulate a systemic inflammatory response. This review aims to summarise the current evidence for the pathogenesis of HE from the clinical perspective of infection and inflammation and the synergistic relationship with ammonia, with consideration of how gut dysbiosis and intestinal barrier dysfunction contribute.

**Table 1:** Westhaven (WHC) and International Society of Hepatic Encephalopathy and Nitrogen Metabolism (ISHEN) criteria describing severity of HE (Vilstrup et al. 2014)

WHC Grade	ISHEN criteria	Description
Minimal/0	Covert	Abnormal results of established psychometric or neuropsychological tests without clinical manifestations
1		Trivial lack of awareness, euphoria or anxiety, shortened attention span, impairment of addition or subtraction, altered sleep rhythm
2	Overt	Lethargy or apathy, disorientation for time, obvious personality change, inappropriate behaviour, dyspraxia, asterixis
3		Somnolence to semi stupor, responsive to stimuli, confused, gross disorientation, bizarre behaviour
4		Coma

## Systemic inflammation and infection

### Systemic inflammatory response syndrome

Over 30 years ago the systemic inflammatory response syndrome (SIRS) was proposed to describe the immune response to infection (termed sepsis) and aseptic aetiologies such as pancreatitis, ischaemia, trauma, haemorrhagic shock, and immune mediated injury (Bone et al. 1992). The hyperinflammatory state seen in acute liver failure or alcohol-associated hepatitis driven by a systemic immune response to hepatocyte death can be likened to the SIRS response. Diagnostic criteria for SIRS included two or more of the following: 1) Body temperature > 38 ℃ or < 36 ℃; 2) heart rate > 90 beats per minute, 3) respiratory rate > 20 breaths per minute or hyperventilation indicated by pCO2 < 32mmHg; 4) a white blood cell count > 12,000/cu mm, or < 4,000/cu mm or > 10% immature bands (Bone et al. 1992). Since the advent of SIRS, various other diagnostic and prognostic scoring systems, such as the updated sequential organ failure assessment (SOFA) and quick SOFA (qSOFA) scores have been introduced to clinical practice (Singer et al. 2016). Whilst previous definitions have viewed sepsis on a continuum of severity from sepsis (SIRS with infection) to severe sepsis (sepsis with evidence of acute organ dysfunction) to septic shock (hypotension after fluid resuscitation), more recent definitions have abandoned the traditional description of infection with SIRS to employ a definition that acknowledges the multi-organ involvement of the inflammatory response and the significant mortality associated with this (Bauer et al. 2020). The latest iteration of guidelines defines sepsis as “life-threatening organ dysfunction caused by a dysregulated host response to infection” (Singer et al. 2016). Regardless of updated clinical definitions, our understanding of the pathophysiology of SIRS to an insult remains very relevant due to its incorporation in the methodology of studies performed within the last two decades.

On a molecular level, the inflammatory response is triggered by the release of molecules associated with either infective pathogens called pathogen associated molecular patterns (PAMPS), (*e.g.* lipopolysaccharide (LPS), a component of the gram negative bacterial cell wall) (Faure et al. 2001), or damage associated molecular patterns (DAMPS) which are endogenous intra-cellular molecules that are released from damaged host cells through trauma, ischaemia–reperfusion or toxic injuries (Gotts and Matthay 2016). The innate immune system is activated by these molecules through cell surface receptors such as toll-like receptors (TLRs) and NOD-like receptors (Faure et al. 2001). This leads to activation of transcription factor nuclear factor-kappa B (NF-KB) which is responsible for activating genes that encode pro-inflammatory cytokines such as TNF-$\alpha$, interleukin (IL)-1 and IL-6 and IL-1β (Weighardt and Holzmann 2008). This cytokine storm results in direct host tissue injury from reactive oxygen species and complement activation. Endothelial dysfunction occurs with increased leukocyte adhesion, production of nitric oxide (NO), vasodilation and increased endothelial permeability resulting in tissue oedema and circulatory collapse (Aird 2003). Other epithelial barriers are affected including the alveolar epithelial barrier resulting in acute respiratory distress syndrome (Matthay et al. 2012), as well as the intestinal epithelial barrier resulting in bacterial translocation, creating a vicious cycle of gut-derived PAMPs entering the circulation (Fink 2003). Multi-organ failure then ensues with acute kidney injury, encephalopathy, immune paresis and disseminated intravascular coagulopathy. There also exists a compensatory anti-inflammatory response syndrome associated with raised levels of anti-inflammatory mediators IL-4, IL-10 and TGFβ (Döcke et al. 1997; Antoniades et al. 2008).

### Systemic inflammation, infection and hepatic encephalopathy in acute liver failure

The association of systemic inflammation with brain dysfunction is by no means a new concept. This dates back over 2500 years to Hippocrates of Kos (460–370 BCE) who described patients with abscesses and fever as having ‘phrenitis’ or inflammation of the mind (Chadwick and Mann 1950, pp 50, 223). Later, in 200 CE Claudius Galen offered the first description of delirium, suggesting inflammation affected the mind ‘sympathetically’ (Mendez 1996, pp 29–38) and much later in the nineteenth century Sir William Osler described septicaemia where “organisms enter the blood from some local septic focus” being associated with impaired brain function in the form of “early delirium or marked mental prostration and apathy” (Osler 1892, pp114-118).

In acute liver failure, an association of inflammation with HE has long been demonstrated through correlation with SIRS (Rolando et al. 2000; Vaquero et al. 2003; Jalan et al. 2004a, b, Miyake et al. 2012). Rolando et al. were able to show that acute liver failure patients with a higher SIRS score on admission to the intensive care unit were more likely to develop worsening encephalopathy, raised intracranial pressure and death. This was regardless of a diagnosis of infection (Rolando et al. 2000). In their prospective observational study, Vaquero et al. demonstrated a temporal association between the SIRS score and progression of hepatic encephalopathy. Again, this effect was independent of the development of infection (as defined in this study by the presence of bacteraemia). However, this was only true of the non-acetaminophen-induced acute liver failure group as the development of infection in the acetaminophen-induced acute liver failure group was associated with worsening grades of HE (Vaquero et al. 2003). Jalan et al. measured cerebral blood flow and intracranial pressure longitudinally in a group of patients with grade 4 HE with a poor prognosis, as defined by the King’s College Criteria, requiring intubation and ventilation (Jalan et al. 2004a, b). They found that the group of patients with raised ICP > 20mmHg had a significantly higher SIRS score, as well as significantly greater serum concentrations of IL-1β, IL-6 and TNFα, with TNFα concentrations correlating directly with increased cerebral blood flow (Jalan et al. 2004a, b).

Due to the size of peripherally derived pro-inflammatory cytokines, it was originally thought that they were too large to permeate the blood brain barrier (BBB). Various studies have attempted to identify the mechanism of signalling that results in neuroinflammation thought to underpin the pathology of HE in acute liver failure (Wright et al. 2007; D'Mello et al. 2009). Astrocytes are supportive glial cells in the central nervous system (CNS) that play an essential role in BBB function as well as trophic and metabolic support for neurones. Post mortem examination of brain tissue in acute liver failure reveals cerebral oedema caused by a cytotoxic insult with evidence of astrocyte swelling and a disrupted BBB (Kato et al. 1992). As demonstrated in studies of the neuroinflammatory effects of sepsis, permeability of the BBB may be influenced by neuronal stimulation via the vagus nerve (Akrout et al. 2009). Secondary messengers produced in response to cytokines such as nitrous oxide and prostaglandins or TNFα may act directly to modulate BBB permeability (Akrout et al. 2009; D'Mello et al. 2009). More recent work has focussed on microglial cells which are innate CNS immune cells that normally reside in a quiescent state in the brain unless ‘activated’ by an inflammatory stimulus (Hoogland et al. 2015). In murine models of acute liver failure, microglial activation which is indicative of neuroinflammation, has been repeatedly demonstrated (Jiang et al. 2009a, b; Jiang et al. 2009a, b; McMillin et al. 2019), but to date, few studies have been able to reproduce these findings in humans (Zemtsova et al. 2011; Dennis et al. 2014). In their murine model of hepatic injury using bile duct ligation, D’Mello et al. demonstrated that fluorescein labelled peripheral monocytes entered the brain. They were able to show that prior to this migration, microglial activation occurred through peripheral TNFα signalling, that was diminished, when anti TNFα treatment was administered to the mice (D'Mello et al. 2009). Wright et al. were able to demonstrate that the brain is able to produce cytokines (IL-1β, IL-6 and TNFα) by taking serum samples from a jugular venous bulb catheter in acute liver failure patients with severe HE in the intensive care unit. There was a significant positive efflux of proinflammatory cytokines in those patients with uncontrolled ICP when compared to those without (Wright et al. 2007). Therefore, it is likely that that either through peripheral signalling from the inflammatory cascade or disruptions to the BBB, an inflammatory process is triggered in the brain affecting microglial cells and astrocytes. Consequently, glial cells are able to amplify this by producing further cytokines in an attempt to maintain homeostasis, and in the setting of acute liver failure, this may lead to astrocyte swelling, cerebral oedema and raised ICP associated with advanced stages of HE.

## The role of ammonia

**Fig. 1:**
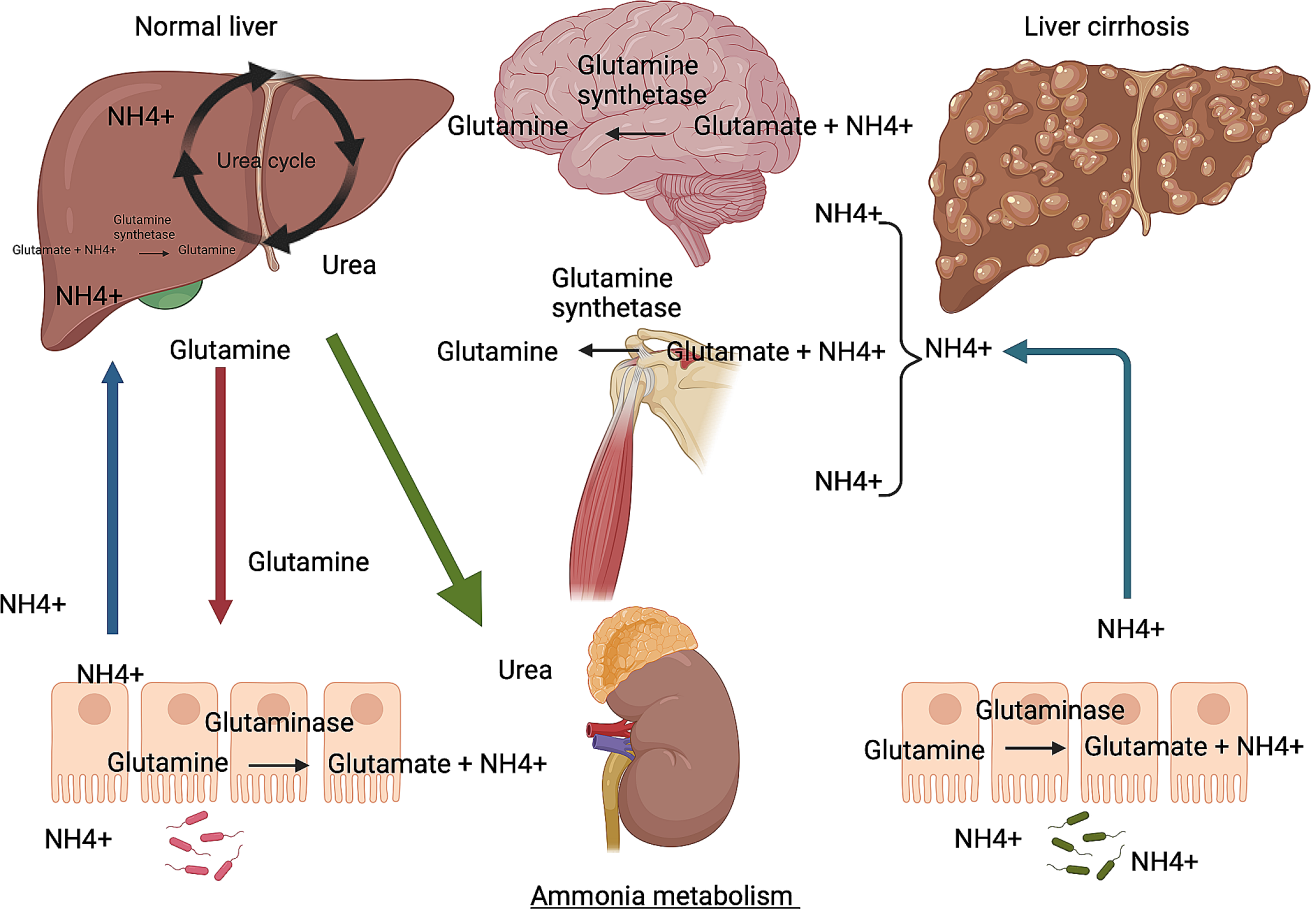
Ammonia metabolism in health and in cirrhosis. Under normal physiological conditions ammonia is produced in the small intestine by enterocytes as a by-product of the catabolism of the circulating amino acid glutamine. Ammonia is also generated by the catabolism of urea by urease producing colonic bacteria after a protein meal. It then enters the portal circulation and is re-cycled back to urea by peri-portal hepatocytes through the urea cycle or converted to glutamine by the action of glutamine synthetase (GS) in peri-venous hepatocytes. Urea is renally excreted once it enters the systemic circulation. In cirrhosis, porto-systemic shunting and reduced hepatocyte metabolic capability result in a reliance on alternative ammonia detoxifying pathways e.g. skeletal muscle. Ammonia is able to permeate the blood brain barrier with astrocytes having the capability to convert it to glutamine through the action of GS. Abbreviations: NH4 +: ammonium ion. Created in Biorender

Under normal physiological conditions ammonia is derived from the gastrointestinal tract (van der Hulst et al. 1997, Olde Damink et al. 2002, van de Poll et al. 2007) and kidneys (van de Poll et al. 2008). In the small intestine it is produced in enterocytes as a by-product of the catabolism of the circulating amino acid glutamine (van der Hulst et al. 1997, Olde Damink et al. 2002, van de Poll et al. 2007). Ammonia is also generated by the catabolism of urea by urease producing colonic bacteria after a protein meal (Walser and Bodenlos 1959; Aoyagi et al. 1966). It then enters the portal circulation and is re-cycled back to urea by peri-portal hepatocytes through the urea cycle, or converted to glutamine by the action of glutamine synthetase (GS) in peri-venous hepatocytes (Häussinger 1986; Shawcross et al. 2023b). Urea is renally excreted once it enters the systemic circulation (Olde Damink et al. 2006). Some ammonia is also metabolised by skeletal muscle which becomes a significant detoxifying source when hepatic reserve is diminished in cirrhosis. Ammonia is able to permeate the BBB with astrocytes having the capability to convert it to glutamine through the action of GS. In liver disease there is reduced hepatocyte metabolic capability and porto-systemic shunting (with or without cirrhosis), which contributes to hyperammonaemia (Khatra et al. 1974) (See Fig. 1).

The central role of ammonia in the pathogenesis of HE originated from the early work of Marcel Nencki and Ivan Pavlov who ran experiments on dogs with portocaval shunts (“Eck’s fistula”) (Nencki et al. 1895; Nencki and Zaleski 1895). These dogs developed neuro-behavioural changes, with evidence of increased urinary ammonia excretion, the theory being, the products of nitrogen substrate degradation (ammonia) absorbed from the gut were directly entering the systemic circulation, without undergoing removal through the hepatic urea cycle (Shawcross et al. 2005). The symptoms experienced by these dogs included aggression, irritability, ataxia and convulsions, culminating in coma as early as 10 days and up six weeks post-surgery. Later experiments demonstrated that these symptoms were exacerbated by a protein meal and the investigators reported increased intracerebral ammonia concentrations, leading to their conclusions about the causative role of ammonia (Shawcross et al. 2005).

In acute liver failure, human clinical studies have reliably demonstrated a correlation with plasma ammonia levels and degree of HE. Plasma ammonia levels predict the development of cerebral oedema, herniation and death (Bhatia et al. 2006; Bernal et al. 2007). Various in vitro studies have provided evidence to suggest a synergistic effect of ammonia and inflammation in brain oedema caused by acute liver failure, with cytokine associated astrocyte swelling potentiated by exposure to ammonia (Chastre et al. 2010, Rama Rao et al. 2010, Rao et al. 2013). Hyperammonaemia induced neuroinflammation has also been demonstrated in rat models of acute liver failure (Rodrigo et al. 2010). Jayakumar et al. were able to demonstrate that this occurred through activation of transcription factor Nuclear Factor-Kappa B (NFKB) in their murine model of acute liver failure (Jayakumar et al. 2011). Endothelial cells at the BBB also have an important role to play. When astrocytes are exposed to the culture medium from brain micro-vessel endothelial cells treated with ammonia, LPS and cytokines, they swell significantly, possibly through the activation of NFKB and TLR-4 (Jayakumar et al. 2012, 2014). Brain oedema is prevented in TLR-4 knock out mice given toxic levels of acetaminophen or when they are pre-treated with a TLR-4 antagonist (Shah et al. 2013). In further studies examining the roll of immune cell surface receptors in this process, TLR- 9 expression in acetaminophen-induced liver failure has been shown to be mediated by ammonia and IL-8 in a synergistic manner to induce systemic inflammation (Manakkat Vijay et al. 2016). In a TLR9-deficient mouse model, following ammonia acetate injection, brain water, macrophage and T cell cytokine production increased in wild type but not TLR9 knock out mice. ODN2088, a TLR9 antagonist, inhibited macrophage and T cell cytokine production and prevented an increase in brain water (Manakkat Vijay et al. 2019).

## Systemic inflammation, infection and hepatic encephalopathy in cirrhosis

In cirrhosis, the role of ammonia is less clear and clinical and mechanistic studies point towards a synergistic effect primarily modulated by the presence of inflammation. Numerous clinical studies have demonstrated that ammonia has a pathogenic role to play in the development of cirrhosis related HE, with those patients with more severe grades of HE exhibiting higher serum ammonia levels (Kramer et al. 2000; Nicolao et al. 2003; Ong et al. 2003; Jain et al. 2012). However, serum ammonia can be elevated in patients with no clinical evidence of overt HE. This is demonstrated in the study by Ong et al. where 121 patients with cirrhosis admitted to hospital were assessed for HE and had ammonia levels taken on admission. Despite increasing ammonia levels correlating with increasing grade of HE, 69% (20/29) of those with no clinically observed HE had an ammonia level above the threshold of normal (Ong et al. 2003). In addition, a significant number of patients with grade 3–4 HE had normal or mildly elevated ammonia levels. It is also worth noting that serum ammonia levels do not immediately normalise after resolution of HE, limiting its clinical utility as a diagnostic biomarker of HE or marker of response to treatment (Nicolao et al. 2003). Odeh et al. showed a strong correlation of TNFα with ammonia levels, increasing with grades of HE. However, they also noted significant overlap between groups and the presence of normal values in patients with HE (Odeh et al. 2005). A clinical study examining 100 patients with severe HE (Westhaven grades 3 and 4) requiring admission to the intensive care unit demonstrated that patients with a higher SIRS score were significantly more likely to have a higher grade of HE and this effect was again independent of the presence of positive bacterial culture. In addition, there was no difference in serum ammonia levels between the groups with grade 3 and 4 HE (Shawcross et al. 2011). Manzhalii et al. have more recently demonstrated a clear correlation with increases in inflammatory cytokines IL-1β, IFN-γ, IL-6 and IL-4 with increasing HE grade in patients with Child–Pugh B cirrhosis as well as in their rat model when compared with controls (Manzhalii et al. 2019). Despite controversies surrounding the clinical utility of ammonia in cirrhosis, Ballester et al. have recently shown in their multi-centre prospective study of clinically stable cirrhotic outpatients, that serum ammonia levels, when standardised to the upper limit of normal, can be used to predict those at highest risk of developing overt HE. Ammonia performed better than the psychometric hepatic encephalopathy score (PHES) or critical flicker frequency test on multivariable analysis, and the team developed a prediction model that also included sex, diabetes, albumin and creatinine (Ballester et al. 2023).

In the study of cirrhosis patients with minimal HE, markers of systemic inflammation have been shown to be higher than those without it (Shawcross et al. 2007). A small but illuminating clinical study demonstrated the synergistic effect that ammonia likely has with inflammation to cause minimal HE (Shawcross et al. 2004). Ten patients with cirrhosis admitted to hospital with SIRS secondary to infections were given an oral amino-acid solution to mimic the nitrogenous dump from a gastrointestinal bleed. Neuropsychological testing was performed before administration of the solution and at 2 and 4 h after administration. Neuropsychological testing was also performed after resolution of infection. In addition, ammonia and inflammatory markers IL-1β, IL-6 and TNFα were taken just before administration of the solution and on resolution of infection. Ammonia levels were performed at 2h and 4h after ingestion of the solution. There was a significant decrease in neuropsychological test performance associated with induced hyperammonaemia at 2h and 4h after administration of the amino acid solution in the infected/inflamed state but a significant improvement in test scores following the resolution of infection and SIRS. There was a significant reduction in peripheral inflammatory cytokines on resolution of the infection, but there was no significant difference in serum ammonia levels when comparing values after administration of the amino acid solution and following resolution of SIRS (Shawcross et al. 2004). This suggests that there is an adverse neurocognitive response to hyperammonaemia only in the presence of inflammation.

Mechanistic studies to explain how chronic hyperammonaemia and peripheral inflammation induce neuroinflammation and how this subsequently leads to impaired cognitive and motor function are abundant (Hernández-Rabaza et al. 2016, Balzano et al. 2020). *In vitro* (astrocytes exposed to ammonia) and animal models of HE show Alzheimer type II astrocytosis as the characteristic pathological lesion in HE, thought to result from mild swelling caused by the osmotic effects of intra-cytosolic glutamine (Gregorios et al. 1985, Norenberg 1987), as well as other ammonia-related changes related to glial fibrillary acidic protein (Bélanger et al. 2002) and peripheral type benzodiazepine receptors (Lavoie et al. 1990). However, the clinical significance of this is uncertain and may represent a slightly dated view of what is likely to be a complex interaction between ammonia, the peripheral immune system, the BBB and astrocytes and microglial cells. Human cerebrovascular endothelial cells exposed to TNFα manifest increased capacity for the transport of ammonia (Duchini et al. 1996). Mangas-Losada et al. demonstrated in patients with minimal HE without any clinical evidence of recent or current infection, that there was selective activation of CD4+CD28- T lymphocytes and differentiation to Th follicular, Th22 and Th17 cells which promote enhanced cytokine production. This was not seen in cirrhotic patients without minimal HE and was independent of degree of liver damage as measured by transaminases (Mangas-Losada et al. 2017). CD4+CD28- lymphocytes are able to directly lyse endothelial cells (*in vitro*) and damage tissues as they express perforin and granzyme B (Nakajima et al. 2002; Dumitriu et al. 2012; Dumitriu 2015; Maly and Schirmer 2015), and they are known to express CD161 which is a molecule that facilitates trans-endothelial migration and tissue invasion (Warrington et al. 2001). Given CD4+CD28- T cells have been shown to infiltrate brain tissue in patients with multiple sclerosis (Broux et al. 2012), the authors postulate a similar mechanism of neuroinflammation in minimal HE (Mangas-Losada et al. 2017). Balzano et al were able to show meningeal T lymphocyte infiltration (Tfh, T17 and some CD4+28- lymphocytes) in post mortem examination of the cerebellar tissue of patients with steatohepatitis. Interestingly, the effect was reduced in the cirrhosis and HE samples. However, the associated microglial and astrocytic activation with loss of Purkinje and granular neurons remained elevated with increasing severity of liver disease (Balzano et al. 2018). Previous work by the same group has demonstrated the combination of hyperammonaemia and inflammation in patients with steatohepatitis is associated with cognitive impairment as determined by the PHES battery of psychometric tests (Felipo et al. 2012).

## Infection and immune dysregulation

Where infection is concerned, Merli et al. examined 150 inpatients with cirrhosis and assessed them for the presence of HE and infection. Overt or minimal HE was identified in 79% of cirrhotic patients with infection without SIRS, in 90% with sepsis, and 42% without any evidence of infection. Interestingly, ammonia levels were significantly lower in the 17 patients with overt HE and infection compared with the 15 patients with overt HE without infection. There was a strong correlation between CRP and psychometric test scores with both improving once the infection resolved. Other markers of inflammation such as inflammatory cytokines were not measured in this study (Merli et al. 2013). This suggests that perhaps the pathogenic mechanisms behind infection driven HE are independent of ammonia.

In acute liver failure, neutrophils have reduced phagocytic activity, increasing susceptibility to bacterial infection (Taylor et al. 2013). Ex vivo studies of neutrophils taken from cirrhotic patients with induced hyperammonaemia using amino acid solution demonstrate significantly reduced phagocytosis compared to cirrhotic control patients given a placebo (Shawcross et al. 2008). In addition, when neutrophils from healthy individuals are incubated in ammonia solution, they demonstrate impaired phagocytosis, increased swelling and spontaneous oxidative burst. A hyponatraemic cellular environment exacerbated these findings and they were ameliorated by the p38^MAPK^ agonist isoproterenol. p38^MAPK^ is part of an intracellular signalling mechanism involved in cell volume regulation (vom Dahl et al. 2001; Shawcross et al. 2008; Shawcross et al. 2010). Spontaneous oxidative burst and reduced phagocytic capacity has been associated with a significantly increased risk of infection, organ failure and mortality (Mookerjee et al. 2007). Alabsawy et al. demonstrated that overt HE among cirrhotic patients with acute decompensation was an independent risk factor for the development of de novo infections (Alabsawy et al. 2022). Serum ammonia was not measured in this study. However, it suggests that the inflammatory state associated with overt HE is particularly immune incapacitating.

## Gut-derived systemic inflammation

**Fig. 2:**
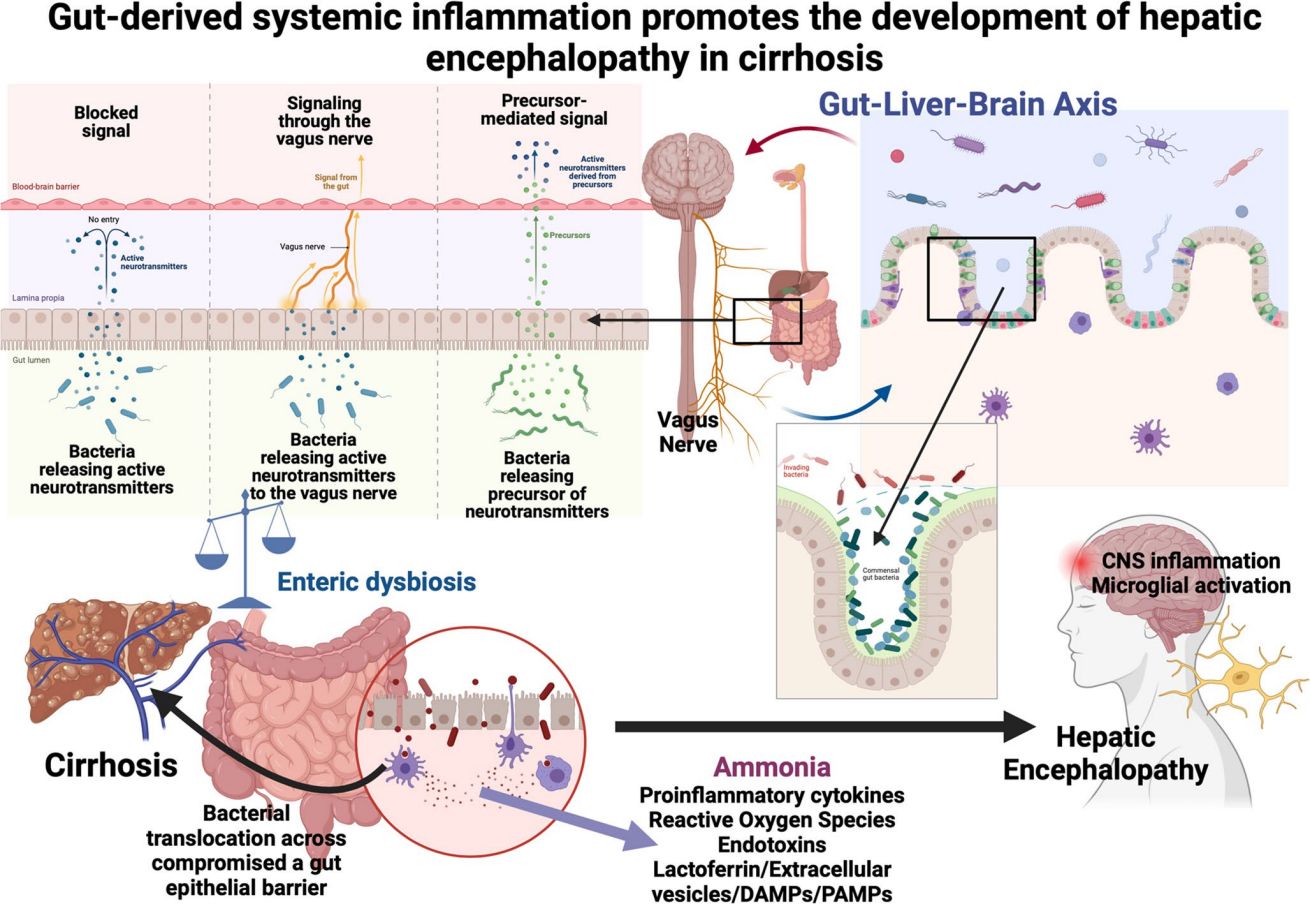
The gut-brain-liver axis. In health, the intestinal barrier is protected and integrity is maintained by commensal gut bacteria. Cirrhosis-associated enteric dysbiosis results in overgrowth of pathobionts which drive gut inflammation, damage the intestinal barrier and enhance ammonia production. This results in translocation of bacteria and PAMPS into the systemic circulation which interact with the innate immune system to cause up-regulation of pro-inflammatory cytokines, generation of reactive oxygen species, release of DAMPS, endotoxins and other pro-inflammatory proteins. This, along with the synergistic effect of ammonia result in inflammatory signals reaching the brain, causing microglial activation and hepatic encephalopathy. Permeability of the blood brain barrier that results in microglial activation may be influenced by neuronal stimulation via the vagus nerve or secondary messengers produced in response to cytokines such as nitric oxide and prostaglandins that are a direct result of gut-derived systemic inflammation. Abbreviations: PAMPS: Pathogen associated molecular patterns; DAMPS: Damage associated molecular patterns. Created in Biorender

The increased peripheral inflammation seen in patients with chronic liver disease is predominantly derived from translocation of PAMPS such as LPS, endotoxin and bacterial DNA through the intestinal epithelial barrier. These molecular products bind to pathogen recognition receptors to initiate the release of various inflammatory cytokines (as suggested in the sepsis model described earlier). A number of mechanisms are thought to influence the rate of translocation that occurs at this barrier which is heavily influenced by the intestinal microbiota, which in cirrhosis exhibits lower diversity and an excess of pathobionts. In addition, studies have demonstrated that patients with cirrhosis also have small intestinal bacterial overgrowth (Bauer et al. 2001). A number of studies have identified specific bacterial organisms associated with HE. Bajaj et al. identified worsening ‘dysbiosis’ as measured by their cirrhosis dysbiosis ratio (CDR) which reflects an arbitrary ratio of autochthonous versus potentially pathogenic bacteria with an increase in Enterobacteriaeceae after the development of HE, despite the use of the prebiotic lactulose in these patients (Bajaj et al. 2014). Alcaligenaceae and Porphyromonadaceae have been associated with worsening cognitive function in patients with cirrhosis (Bajaj et al. 2012). Ruminococcus and Clostridium XIVb in stool and saliva are associated with good cognitive function in patients with cirrhosis on investigations with psychometric testing (Bajaj et al. 2019b). Autochthonous taxa produce short chain fatty acids such as butyrate that are known to maintain the integrity of the intestinal barrier. They also produce antibacterial peptides, nourish colonocytes and compete with pathogenic bacteria for nutrients. Overgrowth with pathogenic taxa such as Enterobacteriaceae result in worsening endotoxaemia and increased peripheral inflammation. Liu et al. were able to demonstrate the direct influence of intestinal dysbiosis on neuroinflammation in their germ-free murine model (Liu et al. 2020). Germ-free mice who received faecal microbial transplantation (FMT) from patients with cirrhosis demonstrated higher degrees of neuroinflammation and activation of GABAergic and neuronal activation compared to those that received FMT from healthy donors. The use of FMT from donors with cirrhosis who themselves had received FMT also exhibited comparably reduced neuroinflammation. The levels of neuroinflammation in the germ-free mice receiving FMT from cirrhotic patients was similar to the neuroinflammation seen in their cirrhotic murine model using carbon tetrachloride (Liu et al. 2020) (Fig. 2).

## Therapeutic targets in HE

### Current treatments

Currently, licenced treatments for HE are mostly targeted at ammonia reduction, primarily through manipulation of gut microbiota. Non-absorbable disaccharides *i.e.* lactulose have been used to treat HE from as early as 1966 (Bircher et al. 1966). The primary mechanism of action of lactulose to reduce HE is through its gut acidifying properties. The sugar compound passes through the gut unabsorbed and unaltered until it reaches the colon where it is metabolised by colonic bacteria into lactic and acetic acids (Bircher et al. 1966). This acidic colonic environment converts free ammonia to ammonium which cannot be reabsorbed across the gut lumen (Elkington et al. 1969). In addition, the acidity favours non-urease producing Lactobacillus, and lactulose functions as a prebiotic for the gut microbiota, increasing their abundance (Bothe et al. 2017). A minor beneficial effect is also derived from the laxative effect of its hyperosmolarity which contributes to reduced ammonia absorption and increased expulsion of nitrogenous compounds in the stool. The beneficial effects on outcomes including a reduction in the risk of developing liver-related complications and mortality in treatment of overt HE and prevention have been demonstrated on meta-analysis (Gluud et al. 2016). Rifaximin, a poorly absorbed and therefore gut selective broad-spectrum antibiotic is another well-established treatment for HE (Bass et al. 2010). The most recent Cochrane meta-analysis has confirmed its effectiveness in the treatment and prevention of HE with little evidence of an effect on overall mortality unless combined with non-absorbable disaccharides (Zacharias et al. 2023). Mechanistic studies show that the antibiotic reduces endotoxaemia in the setting of minimal HE (Bajaj et al. 2013), and reduces systemic inflammation and risk of infection and promotes gut barrier repair (Patel et al. 2022). Aside from demonstrating clear improvements in cognitive function on testing in those with minimal HE, and prevention of HE in the treatment group, Patel et al. demonstrated reduced TNFα, IL-10 and TLR-4 levels in the group of rifaximin-treated cirrhotic patients compared with placebo. This was thought to occur as a result of the supressed oralisation of the gut microbiome, in particular, of species known to produce mucin-degrading enzymes which induce gut barrier damage (Patel et al. 2022). Of note, there was no change in ammonia levels between the treatment and placebo groups (Patel et al. 2022).

### Ammonia and nitrogen scavengers

L-ornithine L-aspartate (LOLA) is an established ammonia lowering treatment made up of two endogenous amino acids thought to exert its effect through removal of ammonia through stimulation of hepatic urea synthesis and glutamine synthesis in skeletal muscle (Kircheis and Lüth 2019). Goh et al. concluded in their Cochrane meta-analysis of the drug’s use in cirrhosis that there was insufficient evidence to support or refute the claims of efficacy in reducing mortality or HE due to the majority of trials graded at high risk of bias (Goh et al. 2018). A more recent meta-analysis assessing efficacy in minimal HE demonstrated efficacy in reversal of minimal HE and preventing progression to overt HE, but there was no effect on mortality compared with placebo or no intervention. Six RCTs were included in the analysis and all were deemed to have a high methodological quality according to the modified Jadad scale (He et al. 2024). Other pharmacological agents that target ammonia reduction include the nitrogen scavenging drugs glycerol phenylbutyrate, polyethylene glycol and sodium benzoate. In a Cochrane meta-analysis all drugs lowered blood ammonia levels versus placebo, but none lowered ammonia compared to non-absorbable disaccharides. Only glycerol phenylbutyrate showed a beneficial effect versus placebo on treating HE and so did polyethylene glycol when compared to lactulose. Even so, the authors were uncertain of the results due to low numbers of trials, insufficient data and bias introduced from pharmaceutical funding of the studies (Zacharias et al. 2019). In the management of acute liver failure, continuous renal replacement therapy has been shown to significantly reduce ammonia levels and improve 21-day transplant free survival compared to intermittent renal replacement therapy or no-renal replacement therapy (Cardoso et al. 2018).

### Treatments specifically targeting the gut microbiota

Probiotics consist of commercially available human derived microbial organisms, that when ingested improve intestinal microbial balance and interact with the host gut microbiota to promote the production of molecules that favourably support intestinal barrier function. A recent meta-analysis on the efficacy of these products in minimal HE demonstrated greater efficacy in reversal of minimal HE and reduction of serum ammonia levels compared to placebo or no treatment. However, they were no more efficacious than lactulose or LOLA (Wibawa et al. 2023). Faecal microbiota transplantation (FMT) involves transplanting stool from healthy donors into patients with gut dysbiosis. Historically this has been used to treat recurrent *Clostridioides difficile* infection (Agrawal et al. 2016), but more recently, the application of FMT has expanded to use in cirrhosis, where the importance of the gut-liver-axis is becoming more apparent (Shawcross et al. 2023a). There have been a number of early phase randomised controlled trials evaluating the efficacy of FMT for the treatment of HE which show promising results (Bajaj et al. 2017a; Bajaj et al. 2019b; Bajaj et al. 2019c; Bloom et al. 2022). In addition, FMT is on the whole safe and well tolerated with mild and transient gastro-intestinal side effects reported such as diarrhoea and abdominal discomfort (Marcella et al. 2021). Serious adverse events are rare (1.4%) (Marcella et al. 2021), and related to upper gastrointestinal tract administration via endoscopy, which is becoming less relevant due to the development of freeze-dried capsule delivery systems. There is one report of a patient dying from drug resistant *Escherichia coli* bacteraemia assumed to be transmitted from FMT (Marcella et al. 2021).

### Targeting systemic inflammation

Human clinical trials using pharmacological agents that directly target systemic inflammation for the treatment of HE are significantly lacking. The majority of data supporting this therapeutic target remain confined to animal models. Despite the repeated demonstration of the role of TNFα in HE, to date, no randomised controlled trials have been undertaken to investigate the use of medications that attenuate this inflammatory cytokine. Numerous murine models have demonstrated effective reversal of the neurocognitive effects of liver-related inflammation with either the use of anti-TNF monoclonal antibody infliximab (Dadsetan et al. 2016a, b; Dadsetan et al. 2016a, b); TNFα neutralising molecule etanercept (Chastre et al. 2012); or animal models that utilise TNFα receptor gene knock down (Bémeur et al. 2010). Only anecdotal evidence exists in the form of case reports for the use of etanercept in 3 cases of poison-induced acute hepatic injuries in China (Xing and Zhu 2021). Minocycline, a tetracycline antibiotic has been shown to prevent microglial activation in LPS injected murine models of acute liver failure (Jiang et al. 2009a, b; Gamal et al. 2014). Non-steroidal anti-inflammatory drugs such as ibuprofen which target cyclooxygenase and other promoters of the inflammatory response have shown promise in animal models but are limited by their relative contra-indication in decompensated cirrhosis due to their effects on renal perfusion and subsequent function (Cauli et al. 2007; Rodrigo et al. 2010). Angiotensin II receptor antagonists have been shown to reduce liver enzymes, ammonia and TNFα levels as well as histological parameters such as peri-portal fibrosis and portal vessel congestion in rat models. However, as with ibuprofen, their use is limited in cirrhosis due to the potential nephrotoxic effects (Murad et al. 2017). The phosphodiesterase-5 selective inhibitor sildenafil has been shown to decrease microglial activation, IL-1β and TNFα levels in the cerebellum and hippocampus in rats with HE compared to rats without HE. This correlated with improved motor co-ordination and enhanced memory and special learning (Hernandez-Rabaza et al. 2015, Agusti et al. 2017). Tadalafil also had promising results in a recent murine model for HE (França et al. 2019). Bicuculline, a GABA receptor antagonist reduced microglial activation, astrocyte activation and IL-1β levels, associated with improved learning index and memory and decreased anxiety-like behaviour in hyperammonaemic rats (Malaguarnera et al. 2019). Albumin has seen a recent resurgence due to the positive results seen in the RELIEF study where albumin dialysis was used via the molecular adsorbent recirculating system (MARS) to treat 189 patients with acute on chronic liver failure. The intervention failed to show any beneficial effect on mortality but did demonstrate significant improvement in HE grades in the intervention group (Bañares et al. 2013). Since then, a meta-analysis has demonstrated a lower pooled risk of overt HE (for treatment and prevention) when given albumin in addition to standard medical treatment, compared to those not given albumin (Teh et al. 2021). A benefit in inpatient mortality but not overall mortality was also seen in the albumin group. Several of the studies included were cohort studies with most studies exhibiting a moderate risk of bias due to selection and attrition bias (Teh et al. 2021). The use of targeted temperature control with moderate hypothermia in the management of acute liver failure has shown promising results for bridging to transplantation and reducing ICP (Jalan et al. 2004a, b). However, a multi-centre randomized control trial assessing the use of treatment at 34°C for 72 h in patients with high grade encephalopathy and ICP monitoring failed to show any benefit in preventing the development of intracranial hypertension or survival compared to management at 36°C (control) (Bernal et al. 2016). The use of hypertonic saline or mannitol in the management of ICP has shown equivalent efficacy in reducing ICP and mortality in acute liver failure, with hypertonic saline less likely to cause a rebound rise in ICP and renal dysfunction than mannitol (Kalal et al. 2022). Finally, Larsen et al. showed that early plasmapheresis using exchange of fresh frozen plasma in patients with acute liver failure of least grade 2 HE improved overall survival compared to standard medical treatment alone. SIRS and SOFA scores were significantly reduced in the plasmapheresis group and they were able to demonstrate lower levels of circulating DAMPs and cytokine production (IL-6, IL-8 and TNF-α) in those receiving plasmapheresis (Larsen et al. 2016). This suggests the clinical benefits may be derived from the treatment’s immune modulating effect and removal of products associated with the innate immune response to hepatic injury.

## Conclusion

Over the last three decades a significant body of evidence has accumulated to support the theory of synergism of inflammation and ammonia in the pathogenesis of HE. More recently, advances in our understanding of gut-derived systemic inflammation and the gut-brain-liver axis has driven advances in new treatment modalities. Currently, these are mainly focussed on targeting gut dysbiosis with FMT and probiotics. However, there remains an untapped area that directly targets peripheral and neuroinflammation and new therapeutic options are desperately needed to treat the debilitating and sometimes fatal complication of hepatic encephalopathy.

## Data Availability

No datasets were generated or analysed during the current study.

## Abbreviations

BBB: Blood brain barrier
CNS: Central nervous system
DAMPS: Damage associated molecular patterns
FMT: Faecal microbiota transplantation
GABA: Gamma-aminobutyric acid
GS: Glutamine synthetase
HE: Hepatic encephalopathy
ICP: intracranial pressure
IL: Interleukin
LOLA: L-ornithine L-aspartate
LPS: lipopolysaccharide
NO: Nitric oxide
NF-KB: Nuclear factor-kappa B
PAMPS: Pathogen associated molecular patterns
PHES: Psychometric Hepatic Encephalopathy Score
qSOFA: Quick SOFA
SIRS: Systemic inflammatory response syndrome
SOFA: sequential organ failure assessment
TGFβ: Transforming growth factorβ
TLR: Toll-like receptors
TNFα: Tumour necrosis factor alpha
